# IgA Vasculitis Across the Ages: Is It Time for a Precision Medicine Approach?

**DOI:** 10.1002/acr2.70083

**Published:** 2025-09-12

**Authors:** A. Gage, R. J. Pepper, J. Marro, A. D. Salama, L. Oni

**Affiliations:** ^1^ Centre for Kidney and Bladder Health University College London London United Kingdom; ^2^ University Hospitals Bristol and Weston NHS Foundation Trust Bristol United Kingdom; ^3^ Centre for Kidney and Bladder Health, University College London and Department of Paediatric Nephrology, Great Ormond Street Hospital for Children NHS Foundation Trust, London, and Department of Women's and Children's Health, Institute of Life Course and Medical Sciences University of Liverpool Liverpool United Kingdom

## Abstract

IgA vasculitis (IgAV; formerly Henoch‐Schönlein purpura) is a systemic small vessel vasculitis most commonly affecting the skin, gut, joints, and kidneys. Nephritis is the most concerning complication for all ages because it carries the risk of progression to irreversible end‐stage kidney failure. The multiorgan nature of the disease, variation in clinical presentation, and unpredictable disease course pose a substantial challenge to timely diagnosis, risk stratification, and a unified approach to management. Precision medicine is defined as a health care approach that uses genetic and molecular profiling alongside phenotypic and environmental data to generate insights to prevent or treat disease. This review article aims to provide an overview of IgAV using the latest literature to highlight three key areas in which precision medicine may have a role in advancing patient outcomes. These three areas are as follows: early phenotyping, risk stratification, and evidence‐based management. Due to the presence of nephritis bringing the greatest risk of morbidity and mortality for patients with this disease, kidney involvement forms the focal point of this review. Like other forms of glomerulonephritis, there are defined stages, from diagnosis to early signs of nephritis, histologically established nephritis, chronic kidney disease, and ultimately kidney failure. Advancing a disease for which there has been very little progress provides a huge opportunity to incorporate precision medicine from the outset to augment traditional monitoring and provide more rapid evidence generation. With transformative treatments on the horizon for IgA‐related glomerular diseases, improving patient outcomes to prevent kidney failure in IgAV is becoming a near reality.

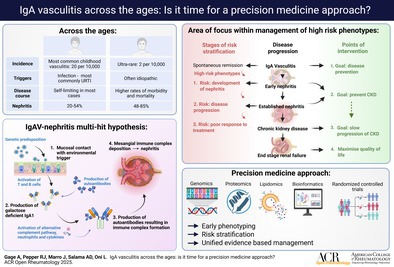

## Graphical abstract

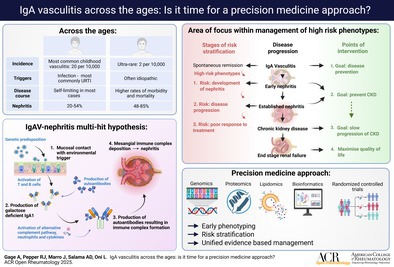

## Introduction

IgA vasculitis (IgAV; formerly Henoch‐Schönlein purpura) is a systemic small vessel vasculitis most commonly affecting the skin, gut, joints, and kidneys.^1^, ^2^ The exact pathophysiology remains an area of ongoing research; however, it is widely acknowledged that the galactose‐deficient version of IgA drives endothelial deposition of IgA‐rich immune complexes, triggering a cascade of inflammatory responses and resulting in a spectrum of organ involvement.^3^, ^4^, ^5^, ^6^

IgAV is the most common childhood vasculitis; however, it can occur at any age.^1^, ^4^, ^7^, ^8^ There are important similarities yet distinct differences in the symptomatic presentation and disease outcomes between pediatric and adult patients. In children, it is largely a self‐limiting phenomenon, whereas the morbidity and mortality rates are significantly higher in adults, in whom presentation is typically later and includes more advanced kidney disease.^9^, ^10^, ^11^ A common theme across the ages is that nephritis is the most concerning complication, bringing a risk of developing chronic kidney disease (CKD) and, in a smaller proportion of patients, progression to irreversible end‐stage kidney failure.^11^ The multiorgan nature of the disease, together with the variation in clinical presentation and disease course, poses a substantial challenge to timely diagnosis, risk stratification, and a unified approach to management.^11^, ^12^ This is compounded by a paucity of high‐quality research in the field, which leads to a strong reliance on expert opinion, resulting in considerable inconsistency in clinical management. Moreover, due to the predominance in the childhood population, IgAV is often considered exclusively a disease of childhood, with few experts in the adult medical field, potentially contributing to a delay in the management of adults.

Precision medicine is a method to incorporate molecular profiling alongside phenotypic and environmental data to generate more accurate insights to prevent or treat disease.^13^ The ultimate aim is to characterize detailed molecular phenotyping of disease that is specific to the individual patient to facilitate a personalized treatment approach. The clinical heterogeneity of IgAV, along with significant evidence gaps to guide the optimum management strategy, lends itself well to embracing implementation of this emerging discipline as the field advances. This review article will outline the characteristics of the condition, with a particular focus on inclusion of patients of all ages, kidney involvement, and the key areas of unmet need in which precision medicine may have a future role.

## Pathophysiology and disease triggers

**Figure 1:**
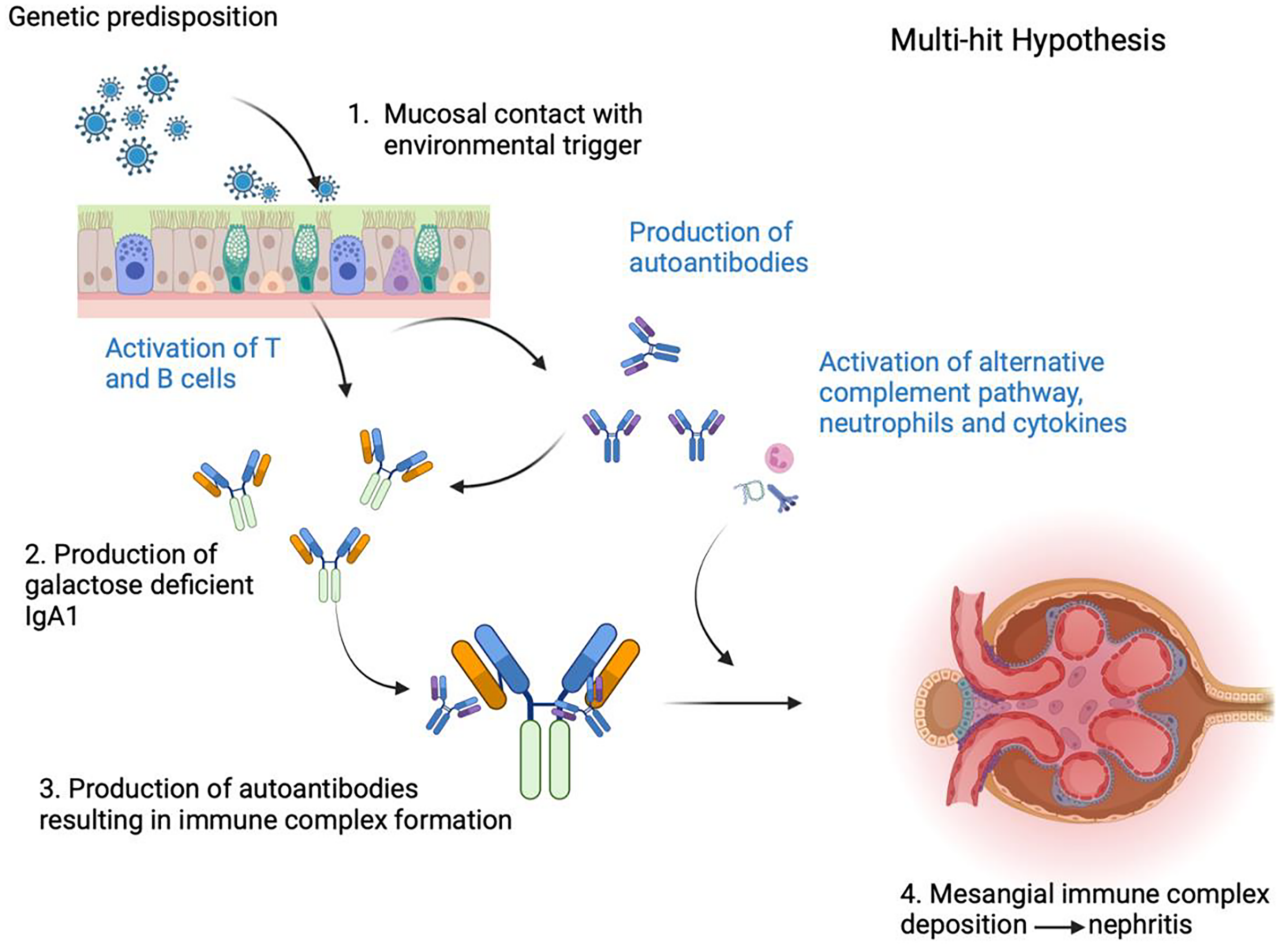
An illustration of the multihit hypothesis demonstrating the relationship between a genetically at‐risk individual and environmental triggers leading to a downstream inflammatory cascade.

IgAV shares many pathophysiologic similarities with the condition IgA nephropathy (IgAN). In contrast to IgAV, IgAN is a renal‐limited condition with an incidence that peaks in young adulthood; more accurate long‐term data and a wealth of treatments are being evaluated.^14^, ^15^ Common to both conditions is the presence of aberrant galactose‐deficient IgA1 (gdIgA1), which is thought to be the key component of a pathologic multihit process^3^, ^4^, ^6^, ^15^, ^16^ (Figure 1). This includes a genetically susceptible individual, environmental triggers, production of gdIgA1, and subsequent activation of inflammatory pathways. Recognized triggers include infections, particularly those affecting the oral or gastrointestinal region where IgA predominates; allergens; and atopy. More recently, environmental pollutants and dietary intake have been identified as potential triggers for this subtype of vasculitis. Gut‐associated lymphoid tissue (GALT) and specifically the Peyer patches in the small bowel seem to be one of the gatekeepers of this pathogenic mechanism.^16^ They function as an antigen sampling system, and they are responsible for producing the most total circulating IgA.^16^ However, mesangial gdIgA1 in IgAV does not contain a secretory component, so it is hypothesized that it is either directly synthesized by the gut mucosa or by GALT‐derived B cells that have migrated to bone marrow.^16^ Heineke et al showed that biopsy samples from patients with IgAN tend to be characterized by glomerulosclerosis and tubular atrophy, whereas those from patients with IgAV are more likely to show endocapillary hypercellularity and crescents.^15^ This finding is consistent with the hypothesis that the final step of the multihit pathway may differ between the two conditions, with a predominance of anti‐endothelial antibodies and neutrophil recruitment in IgAV, perhaps more akin to other forms of vasculitis.^15^

IgAV is associated with an increase in prevalence in spring and winter months, which is most likely due to a rise in seasonal upper respiratory tract infections during these periods.^17^, ^18^, ^19^, ^20^, ^21^ Reports vary, but in pediatric patients, 30% to 50% of cases seem to be preceded by an infection, most commonly *Streptococcus* and tonsillitis,^16^, ^17^, ^18^, ^19^, ^20^, ^21^ whereas idiopathic cases are more common in adults.^11^, ^22^ Observational case studies from the COVID‐19 pandemic suggest that the infectious trigger can also be viral.^20^, ^23^, ^24^ It is uncertain whether there is a relationship with certain vaccinations due to their role in immune system stimulation,^25^, ^26^ and given the biologic association with tonsillitis, the use of tonsillectomy has been proposed as a method to manage disease activity, yet the invasive nature of this procedure limits routine use.^27^, ^28^

## Epidemiology

A series of epidemiologic studies have suggested distinct geographic patterns of disease, with increased prevalence in Southeast Asia aligned with the epidemiology of the similar condition, IgAN.^4^, ^5^, ^19^, ^20^, ^29^, ^30^ In addition, the clinical manifestations of IgAV may vary according to location, with European patients more likely to experience gastrointestinal and musculoskeletal complications and Asian patients more likely to experience genitourinary symptoms.^31^ It is not yet known whether this is due to population‐based genetics or whether other factors are contributing, such as differing dietary or environmental exposures.^32^

In children, IgAV is the most common systemic vasculitis, with around 20 cases per 100,000 per year, and it has a peak presentation between the ages of 4 and 6 years.^5^, ^29^ In contrast, IgAV in adults is a very rare form of vasculitis, and it occurs in around 2 per 100,000 adult population, meeting the definition of an ultrarare disease, with a median age at diagnosis of around 50 years.^5^, ^33^

## Diagnosis

The clinical presentation of IgAV is traditionally described as a tetrad of a characteristic vasculitic rash, abdominal pain, arthralgia, and renal involvement.^1^ For pediatric patients, these are formalized into the EULAR/Paediatric Rheumatology International Trials Organisation (PRINTO)/Paediatric Rheumatology European Society (PRES) classification criteria to aid diagnosis and differentiation from other forms of childhood vasculitis.^34^ They specify the mandatory presence of a purpuric rash affecting the lower legs and buttocks regions, together with the presence of any one of the following: abdominal pain, acute arthritis or arthralgia, proteinuria or hematuria, histopathologic evidence of leukocytoclastic vasculitis, or IgA‐dominant glomerulonephritis.^34^ In patients with adult‐onset disease, there are currently no internationally approved classification criteria, although the authors are aware that European consensus clinical practice recommendations that should meet this need are underway.

## Clinical features

**Figure 2:**
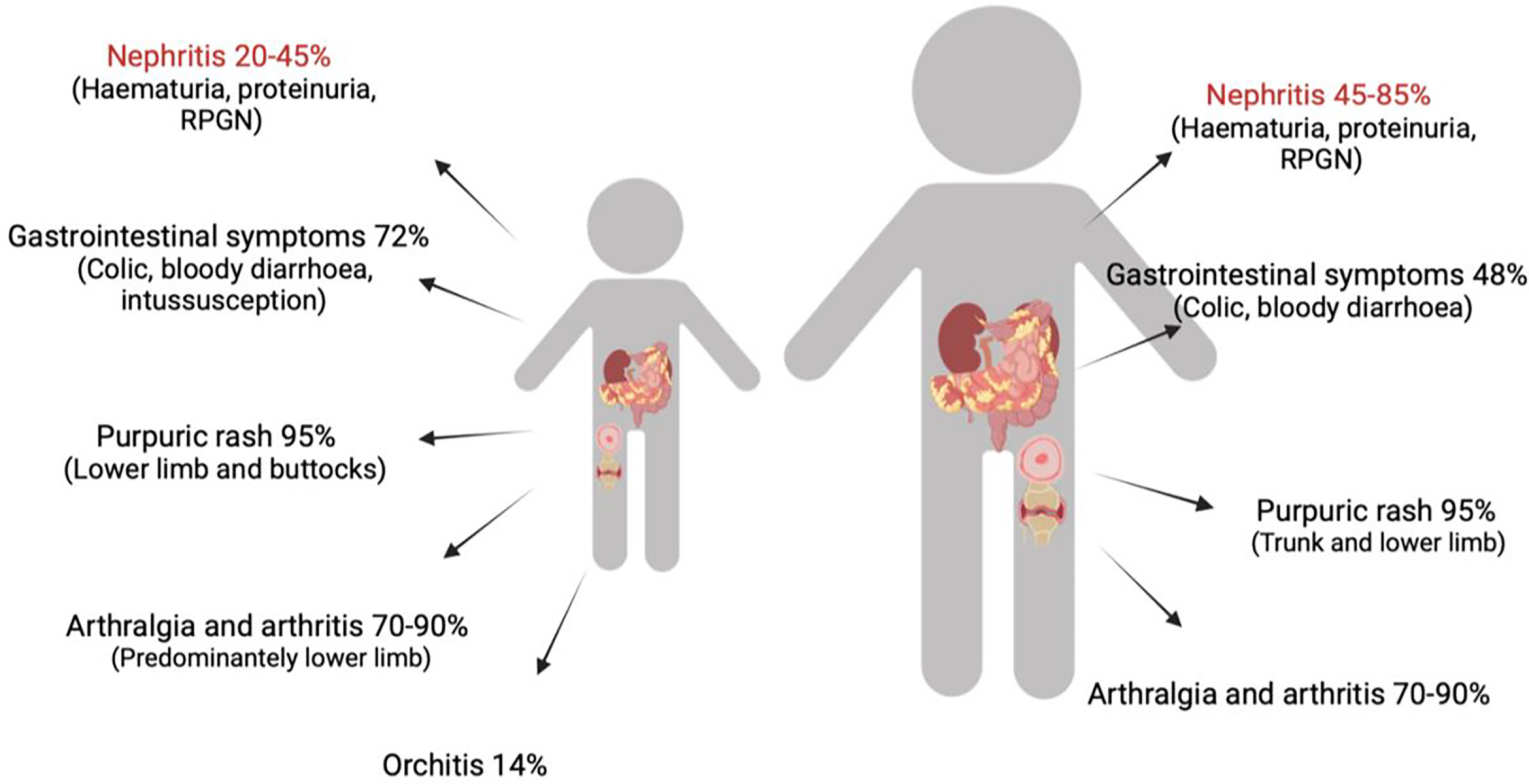
Disease characteristics at presentation reported in the literature in patients with pediatric‐onset disease compared to adult‐onset disease, noting that 95% have a purpuric rash at first presentation, with the remaining 5% developing this mandatory feature subsequently to other symptoms.^12^, ^35^, ^36^, ^37^, ^38^, ^39^, ^40^ RPGN, rapidly progressive glomerulonephritis.

The frequencies of the predominant presenting features of IgAV differ according to age, as shown in Figure 2, with commonality seen in the organs involved.^35^ A purpuric rash is seen in ~95% of patients across all age groups at the point of first diagnosis, at which it almost universally predominantly affects the lower limb and buttocks regions, with extension to truncal and arm involvement, which is more common in adult‐onset disease.^1^, ^10^, ^35^ The rash rarely involves the face, palms, or soles of the feet. A rash can precede the manifestation of other organ involvement by days or even weeks. In the remaining 5%, the vasculitic rash has an onset after other organ manifestations, often abdominal. Polyarthralgia is another common symptom usually present during the acute phase, affecting 70% to 90% children and 61% of adults.^10^, ^36^ Patients tend to report a symmetrical pain involving the knees and ankles that is usually colocated with the vasculitic rash; however, long‐term arthritis and joint damage are extremely rare.^36^

Abdominal symptoms occur more frequently in children than in adults (70% vs 50%) and can be a common reason for pediatric hospital admission during the acute stage of the disease course.^12^, ^35^, ^36^ The symptoms range from fleeting abdominal colic and diarrhea to acute intussusception, bowel infarction, and even gastrointestinal perforation.^37^, ^38^ Intussusception is more common in children, and this is rarely reported in the adult population.^38^ Orchitis is also an important feature, affecting around 14% of boys at presentation, and is often associated with an extensive lower limb vasculitic rash.^37^ Again, this feature is much less frequently reported in adult cohorts. Other, much rarer manifestations include pulmonary hemorrhage, cerebral vasculitis, and neuropathies.^34^

**Figure 3:**
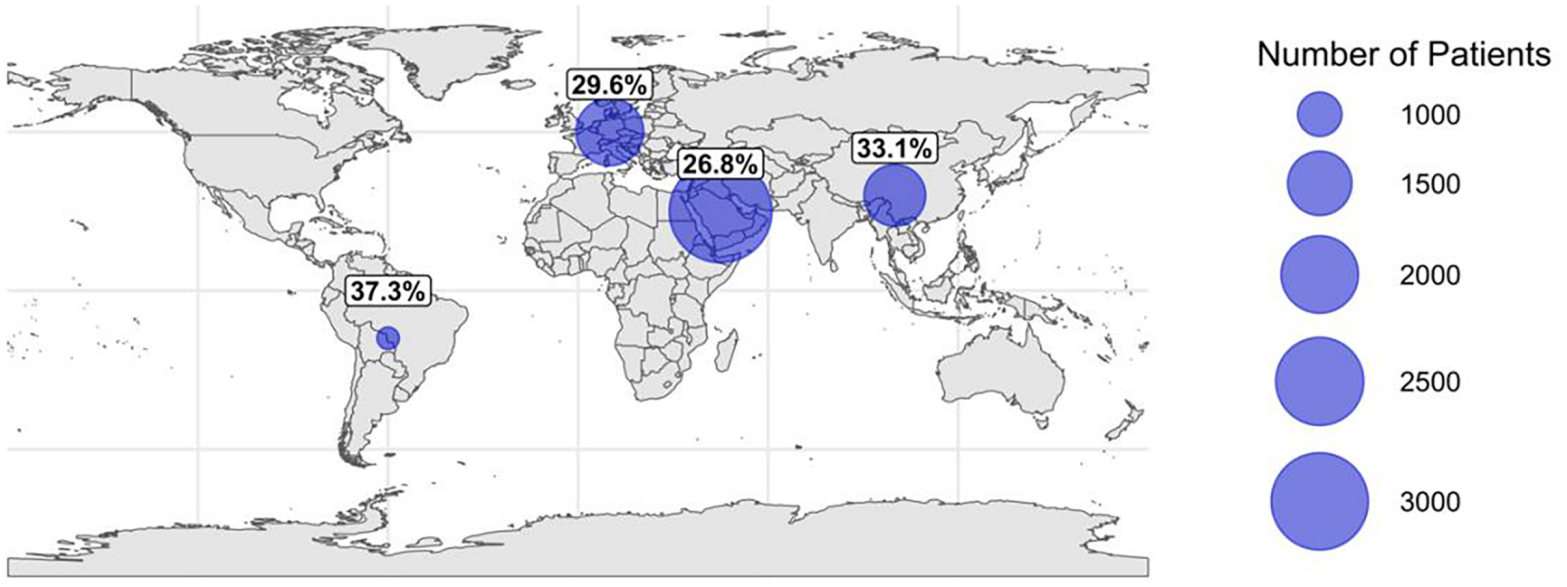
A compilation of the reported rates of nephritis from studies favoring unselected cohorts of children with IgA vasculitis. A total of 24 studies were included (n = 7,180 children). Data are presented as weighted mean rates, with an overall mean incidence of 29.9%. The weighted mean rate for each world region did not significantly differ.

Nephritis in IgAV can occur as both an acute and a subacute complication, and reports demonstrate that it affects 20% to 54% of children and 48% to 85% of adults.^3^, ^11^, ^12^ The most accurate rates of nephritis are reported in childhood cohorts using unselected patients presenting with IgAV. In a scoping literature review performed for this article, 24 studies of unselected pediatric patients involving a total of 7,180 children demonstrated that the mean rate of nephritis in all children with IgAV was 29.9%, and the collated data showed geographic bias in the evidence repartition, highlighting the need for better global representation (refs 19, 39, 40, 41, 42, 43, 44, 45, 46, 47, 48, 49, 50, 51, 52, 53, 54, 55, 56, 57, 58, and Williams C: unpublished observations; Figure 3). In clinical practice, the presentation of nephritis is variable, ranging from microscopic hematuria and proteinuria to macroscopic hematuria, nephrotic‐range proteinuria, hypertension, and acute kidney injury.^1^ Reports vary, but currently around 1% to 8% of pediatric patients and 18% to 23% of adult patients progress to irreversible kidney failure despite treatment.^8^, ^11^

## Management

The mainstay of treatment in the pediatric population is supportive management because IgAV is typically a self‐limiting disease. Rash, arthralgia, and mild abdominal pain are managed with simple analgesia and rarely require further intervention.^59^, ^60^, ^61^ Moderate to severe gastrointestinal, skin, and joint manifestations may benefit from treatment with a short course of glucocorticoids.^62^ The strongest evidence supporting this relates to gastrointestinal involvement, in that several studies have suggested that judicious use of steroids both shortened the length of abdominal symptoms and reduced the incidence of severe complications such as intussusception.^63^, ^64^

There is a paucity of high‐quality trials to inform the most appropriate management of kidney involvement to avoid progression to CKD that has been recognized by serial Cochrane reviews.^65^ Consensus recommendations, including the European SHARE (Single Hub and Access Point for Paediatric Rheumatology in Europe) initiative, are used to guide clinicians; however, they focus only on pediatric patients and remain based on limited evidence.^2^, ^66^ In adults, the Kidney Disease: Improving Global Outcomes (KDIGO) guidelines reflect the paucity of evidence.^67^

Due to the lack of evidence in both the adult and pediatric populations, the management of nephritis relies heavily on expert opinion, introducing variability. Renin–angiotensin inhibition and sodium‐glucose transport protein 2 inhibitors (limited to adult patients at present) are used as standard of care in patients with persistent proteinuria or secondary CKD.^68^ In the early stages of disease, there are randomized controlled trials that show no benefit to using steroids in all patients to try and prevent the development of nephritis.^62^, ^63^, ^69^ However, large cohort studies demonstrate that steroids are commonly used post histological diagnosis—mostly in complex disease, such as cases with nephrotic‐range proteinuria, impaired kidney function, and adverse histological features—together with another immunosuppressive agent, most commonly mycophenolate mofetil (MMF) as adjunctive immunosuppression or as a steroid‐sparing agent and more recently rituximab for B cell depletion.^70^ Rapidly progressive glomerulonephritis (RPGN) and cases of >50% crescents on the kidney biopsy sample are treated similarly to other causes of severe systemic vasculitis, with intravenous immunosuppression consisting of high‐dose steroids, cyclophosphamide, or alternative broad immunosuppressive agents, such as MMF, and rarely there may be consideration of using plasma exchange if there is imminent organ or life‐threatening disease.^10^, ^64^, ^67^, ^71^

In contrast to the management of IgAV, there have been encouraging advances in using targeted management strategies to treat patients with the renal‐limited, IgA‐mediated disease, IgAN, which, as mentioned previously, shares many pathophysiologic similarities with IgAV. One example of an advance is the reported effect of modified‐release budesonide (Nefecon), which targets drug distribution to Peyer patches in the terminal ileum.^72^ The NeflgArd study was a phase 3 trial recruiting 364 adult patients with IgAN to Nefecon or placebo and found a statistically significant reduction in the rate of decline in the estimated glomerular function rate (eGFR) with Nefecon versus placebo (time‐weighted average change of eGFR was −2.47 mL/min/1.73 m^2^ vs −7.52 mL/min/1.73 m^2^) and improvement in proteinuria during a nine‐month treatment period when compared to placebo.^72^ However, the formulation is not suitable for younger children, and therefore translation to IgAV may be challenging. Other agents on the horizon for IgAN that hold promise for use in IgAV nephritis and may not have adverse side effect profiles include avacopan, iptacopan, and novel B cell targets such as anti‐APRIL inhibitors.^64^

## Prognosis

The prognosis of IgAV depends on the age of the patient at presentation and the degree of organ involvement during the disease course. In children, the outlook is very good, with 90% of pediatric cases having resolution of symptoms within one to two months.^12^ As age increases, the proportion of patients who experience treatment dependency or persisting or relapsing disease rises.^7^, ^66^ Reported relapse rates increase from an average of 15% in children to around 30% in adults.^7^, ^11^ Atypical presentations of IgAV in pediatric patients seem to be associated with poorer outcomes.^7^ Both children and adults with nephritis, especially those with persistent proteinuria, impaired kidney function at presentation, and hypertension, have a poorer prognosis, with risk factors for CKD being like those seen in other causes of glomerulonephritis.^11^, ^66^ The risk of death in adults rises with age and is more commonly seen in patients with severe gastrointestinal involvement, RPGN, and pulmonary involvement.^11^ The complications experienced in this disease, along with the lack of evidence‐based treatment options, highlight the unmet need in understanding the disease.

## The opportunity to embrace precision medicine in IgAV

**Figure 4:**
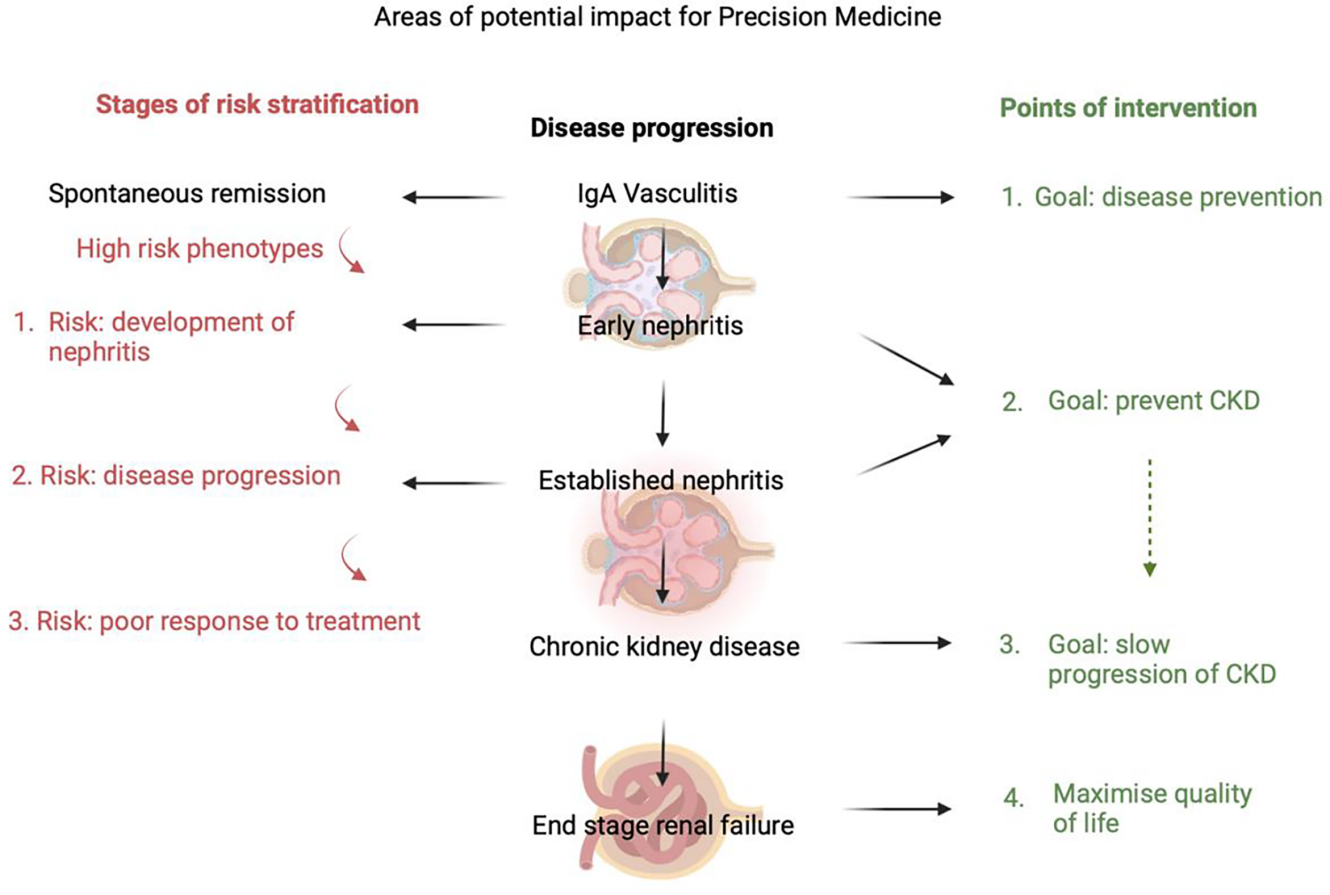
An illustration of the disease progression that identifies stages of potential risk stratification and opportunities for intervention. CKD, chronic kidney disease.

The current era is witnessing a shift from clinically describing diseases to biologically depicting the disease course from diagnosis to established organ failure, with the advent of more specific immune‐based therapy. Precision medicine is an expanding discipline, consisting of “multiomic” approaches (eg, genomics, metabolomics, lipidomics, and proteomics) and data integration to optimize the understanding of disease pathophysiology and management.^13^ Even in other forms of glomerulonephritis, the use of multiomics has yet to reach its full potential, with a recent systematic review demonstrating limited sample sizes for each subtype of glomerulonephritis for proteomics (average sample size n = 35 patients [range 10–103]) and metabolomics/lipidomics (average samples size n = 133 [range 13–487]), hindering positive findings.^73^ The three key areas of unmet need in IgAV in which precision medicine may have a role are early disease phenotyping, risk stratification of disease progression, and an evidence‐based approach to guide individualized management, and each unmet need represents a barrier to advancing the disease. Because nephritis carries the highest risk of morbidity and mortality for patients, it acts as an example to form the focal point of this discussion, with the patient's clinical journey providing key time points for potential intervention (Figure 4).

### Early phenotyping of IgAV nephritis

An accurate diagnosis and classification of a disease is critical to guiding the optimal management of any condition. For pediatric patients, the diagnosis of IgAV is supported by the EULAR/PRINTO/PRES classification criteria,^34^ which demonstrate a sensitivity and specificity of 100% and 87%, respectively.^34^, ^74^ In contrast, there are no validated diagnostic criteria for adult patients with IgAV, and due to the drug development process demanding the need to typically evaluate treatments in adults first, this acts as a major barrier to advancing outcomes for children. There have been studies trialing the extrapolation of the pediatric diagnostic criteria for use in adult‐onset disease; however, they have not been widely adopted, and adults are likely to require specific recommendations.^74^

Genomics is a form of precision medicine. There is a growing body of evidence linking certain genotypes with an increased susceptibility to IgAV; however, there have been no clear causative genetic markers that can direct clinical practice. Genome‐wide associations studies performed in Europe and Asia have consistently identified an increased prevalence of specific HLA single‐nucleotide peptide polymorphisms in patients with IgAV.^75^, ^76^, ^77^, ^78^, ^79^, ^80^, ^81^, ^82^, ^83^ These vary according to patient ethnicity, with HLA–DRB1*01:01, DQB1*05:01, and DQA1*01:01 being the most consistently reported in European and Asian patients.^82^ Non‐HLA genetic risk factors include those associated with the following immune pathway markers: C4 protein, tumor necrosis factor α, 70‐kDa heat‐shock protein member 2 (HSPA2), interleukin‐18 (IL‐18), transforming growth factor β1, the chemokine monocyte chemoattractant protein 1, selectin P, angiotensin, angiotensin‐converting enzyme, CTLA‐4, nitric oxide synthase 2, endothelial nitric oxide synthase, paraoxonase 1, and the MEFV gene.^84^

For validation of any precision medicine techniques, objective monitoring of disease activity is required; however, this is relatively limited because the Birmingham Vasculitis Activity Score and Pediatric Vasculitis Activity Score involved very few patients with IgAV, and thus they may lack the specificity required to truly capture the granular disease course for this form of vasculitis. Specific adaptation of these scoring tools is therefore required to ensure the clinical tools can define disease activity, a key step to integrating precision medicine. Similarly, agreement on optimal histologic classification scoring tools is required, perhaps aligned with those preferred for IgAN.^85^ Higher titers of gdIgA1 have been associated with a more severe disease phenotype in IgAV and specifically an increased risk of nephritis in a few studies^1^, ^3^; however, this is not a consistent finding, and their direct pathogenicity remains unknown because not all patients who develop the disease have high circulating levels of gdIgA1, and controls have been reported with higher than average titers yet no clinical relevance.^86^ Targeting gdIgA1 may be a candidate for intervention if better understanding defines its pathologic role in the early phase of this disease.^86^ Alternative markers include the use of serum CD89–IgA complexes, which have been studied in pediatric patients, in whom higher titers correlated with increased disease severity, and further work is needed to see if this work is reproducible on a larger scale and applicable to patients of all ages.^87^

### Risk stratification of IgAV nephritis

Like many rare inflammatory diseases that involve the kidney, there is a spectrum of disease progression that occurs through defined stages, from the presence of early nephritis to established nephritis, CKD, and then kidney failure. The need for early and accurate phenotyping is essential to incorporate biologic stratification of patients according to their risk of developing severe organ involvement. Currently in clinical practice there is minimal stratification of patients, with the only example being early nephritis monitoring tailored according to the presence of proteinuria. The opportunity to enhance clinical stratification using precision medicine was demonstrated in an article by Kung et al, who performed targeted immune transcript profiling using gene expression analysis in kidney and skin histology in patients with IgAV, IgAN, and IgA‐dominant, infection–related glomerulonephritis.^88^ They revealed that despite the skin biopsy sample being histologically undisguisable using conventional methods, the gene expression profile yielded three targets in patients who developed subsequent nephritis, with significantly decreased skin expression in S100A8, which forms calprotectin; the gene encoding IL‐9; and the killer cell Ig‐like receptor activating subgroup 1 probe, which encodes activating and inhibitor receptors regulating natural killer cell effector function.^88^ Although one study found suggestions of association with more severe gut and renal involvement, the majority have only been able to show susceptibility to disease development rather than a propensity toward a specific phenotype.^75^, ^76^, ^77^ Xu et al^89^ have created a number of genetic risk scores (GRS) to help predict the risk of developing IgAN. They showed a significant correlation between patients with the top 20% of GRS and disease development and also found that those individuals were more likely to have a worse prognosis. An equivalent score for patients with IgAV would be an important move toward informing risk stratification and individualized management for this rare disease.^89^

There is a breadth of work looking at non‐HLA genes, including those related to complement, cytokines, chemokines, and adhesion molecules, and their relationship with nephritis. So far, none of these studies have been conclusive, but they have added to our growing understanding of the pathophysiology of the disease.^41^, ^52^ Due to the direct relevance of urine in its ability to reflect kidney inflammation and the noninvasive nature of this biofluid, there have been many studies focusing on urinary biomarkers to tailor the management of IgAV nephritis. Of note, almost all published studies have been performed exclusively in children. Increased urinary excretion of IgA, IgM, IgA–IgG complexes, serum IgA soluble CD89 complexes, IL‐6, IL‐8, and IL‐10 in patients with IgAV nephritis is a consistent finding across several pediatric studies.^90^, ^91^ Urinary IgA and IgM were commonly found in the highest titers.^90^, ^91^ Berthelot et al found that higher titers of urinary IgA were associated with poorer kidney outcomes at one year and that it was a superior predictor than conventional markers of disease progression (age, eGFR, and proteinuria).^91^ A systematic review of urinary biomarkers in 2017 compared results from 13 eligible studies including 2,446 pediatric patients. It found that the following markers were most promising as early indicators of nephritis and may better reflect disease severity when compared to conventional measures such as proteinuria or kidney function changes: kidney injury molecule 1, monocyte chemotactic protein 1, and *N*‐acetyl‐β‐glucosaminidase.^92^ More work is needed to establish which one, or combination, of these markers is the most reliable reflection of disease activity; whether they are superior to traditional clinical markers of disease activity, such as standard proteinuria; and whether they are applicable to adult patients, in whom there is a much greater risk of CKD progression. Following preclinical discovery and validation, the route to clinical implementation would need to be defined.

### Evidence‐based management of IgAV nephritis

Evidence‐based management of nephritis remains the major weakness in this disease and a key priority for further research. To hasten the drug development process for this condition, the biologic relationship between IgAV nephritis and IgAN would help to evaluate the degree of similarity to establish whether repurposing agents across disease indications would yield opportunity.^93^ The CureGN study, a large multicenter prospective observational cohort study commissioned by the National Institute of Diabetes and Digestive and Kidney Disease, aimed to deepen epidemiologic, clinical, and biochemical correlations of several glomerulopathies, including IgA‐related disease.^94^ From the total, 39.2% of the cohort had IgAV, and they were found to be more likely to have a degree of reversibility regarding urinary sediment and impaired kidney function when compared to patients with IgAN.^94^ This could be due to patients with IgAV presenting with “visible” extrarenal symptoms. The kidney biopsy samples in both diseases show a predominance of mesangial IgA deposition, but additional subendothelial IgA deposits and neutrophilic infiltration are more often seen in IgAV nephritis.^93^, ^95^ NephCure is another large multicenter consortium collecting data from patients with all‐cause nephrotic syndrome with the aim of improving treatment options and outcomes.^96^ The consortium includes patients with IgAV, albeit in small numbers.

Multiple other potential therapeutic targets have also been proposed, including CD89, the alternative complement pathway, and neutrophil extracellular traps.^87^, ^91^, ^97^, ^98^ Currently, these are largely theoretical, based on proposed models of pathophysiology. There are two studies, one using a mouse model and one in vivo, suggesting that neutrophil activity plays an important role in disease activity alongside immune complex formation,^91^, ^93^, ^99^ and this may be a distinguishing feature supporting the need for differing therapeutic approaches to IgAN; however, preclinical models of IgA‐related glomerular diseases do have limitations. There are already several drugs that could be used as targeted agents, including avacopan, iptacopan, sibeprenlimab, and secukinumab, alongside more commonly used B cell–depleting agents, such as rituximab.^100^ Again, further scientific understanding is needed to implement targeted management with confidence.

## Conclusions

IgAV is a neglected rare inflammatory condition in terms of evidence‐based management and is most often seen in a young population, who have a risk of lifelong consequences, yet adults with this condition have increased rates of late recognition and secondary morbidity associated with adult‐onset disease. There is significant variability in current practice, with important missing components to align clinical classification, particularly for adult‐onset disease, which can make advances in precision medicine extremely challenging.

The unmet need in this disease, advances in multiomic approaches, and recognition of the benefits of collaborative working may provide an opportunity to implement precision medicine quicker in IgAV than for established diseases when implementing change can be complex. It is therefore an exciting area in which the early identification of patients with high‐risk phenotypes could completely eliminate CKD in this condition.

## AUTHOR CONTRIBUTIONS

All authors contributed to at least one of the following manuscript preparation roles: conceptualization AND/OR methodology, software, investigation, formal analysis, data curation, visualization, and validation AND drafting or reviewing/editing the final draft. As corresponding author, Dr Oni confirms that all authors have provided the final approval of the version to be published and takes responsibility for the affirmations regarding article submission (eg, not under consideration by another journal), the integrity of the data presented, and the statements regarding compliance with institutional review board/Declaration of Helsinki requirements.

REFERENCES1

Roache‐Robinson
P
, 
Killeen
RB
, 
Hotwagner
DT
. 
*IgA Vasculitis* (*Henoch‐Schönlein Purpura*). StatPearls Publishing; 2024.307259372

Ozen
S
, 
Marks
SD
, 
Brogan
P
, et al. European consensus‐based recommendations for diagnosis and treatment of immunoglobulin A vasculitis‐the SHARE initiative. Rheumatology (Oxford)
2019;58(9):1607–1616.30879080
10.1093/rheumatology/kez0413

Hastings
MC
, 
Rizk
DV
, 
Kiryluk
K
, et al. IgA vasculitis with nephritis: update of pathogenesis with clinical implications. Pediatr Nephrol
2022;37(4):719–733.33818625
10.1007/s00467-021-04950-yPMC84904934

Xu
L
, 
Li
Y
, 
Wu
X
. IgA vasculitis update: Epidemiology, pathogenesis, and biomarkers. Front Immunol
2022;13:921864.36263029
10.3389/fimmu.2022.921864PMC95743575

Piram
M
, 
Mahr
A
. Epidemiology of immunoglobulin A vasculitis (Henoch‐Schönlein): current state of knowledge. Curr Opin Rheumatol
2013;25(2):171–178.23318735
10.1097/BOR.0b013e32835d8e2a6

Song
Y
, 
Huang
X
, 
Yu
G
, et al. Pathogenesis of IgA vasculitis: an up‐to‐date review. Front Immunol
2021;12:771619.34858429
10.3389/fimmu.2021.771619PMC86306197

Marro
J
, 
Williams
C
, 
Pain
CE
, et al. A case series on recurrent and persisting IgA vasculitis (Henoch Schonlein purpura) in children. Pediatr Rheumatol Online J
2023;21(1):85.37580746
10.1186/s12969-023-00872-1PMC104244348

Trnka
P.

Henoch‐Schönlein purpura in children. J Paediatr Child Health
2013;49(12):995–1003.24134307
10.1111/jpc.124039

Tracy
A
, 
Subramanian
A
, 
Adderley
NJ
, et al. Cardiovascular, thromboembolic and renal outcomes in IgA vasculitis (Henoch‐Schönlein purpura): a retrospective cohort study using routinely collected primary care data. Ann Rheum Dis
2019;78(2):261–269.30487151
10.1136/annrheumdis-2018-21414210

Yaseen
K
, 
Herlitz
LC
, 
Villa‐Forte
A
. IgA vasculitis in adults: a rare yet challenging disease. Curr Rheumatol Rep
2021;23(7):50.34196893
10.1007/s11926-021-01013-x11

Stanway
J
, 
Brown
N
, 
Pervez
A
, et al. IgA vasculitis nephritis‐outcomes in adult‐onset disease. Rheumatology (Oxford)
2025;64(2):690–696.38273659
10.1093/rheumatology/keae03012

Oni
L
, 
Sampath
S
. Childhood IgA vasculitis (Henoch Schonlein purpura)‐advances and knowledge gaps. Front Pediatr
2019;7:257.31316952
10.3389/fped.2019.00257PMC661047313

Ginsburg
GS
, 
Phillips
KA
. Precision medicine: from science to value. Health Aff (Millwood)
2018;37(5):694–701.29733705
10.1377/hlthaff.2017.1624PMC598971414

Selvaskandan
H
, 
Shi
S
, 
Twaij
S
, et al. Monitoring immune responses in IgA nephropathy: biomarkers to guide management. Front Immunol
2020;11:572754.33123151
10.3389/fimmu.2020.572754PMC757284715

Heineke
MH
, 
Ballering
AV
, 
Jamin
A
, et al. New insights in the pathogenesis of immunoglobulin A vasculitis (Henoch‐Schönlein purpura). Autoimmun Rev
2017;16(12):1246–1253.29037908
10.1016/j.autrev.2017.10.00916

Barratt
J
, 
Rovin
BH
, 
Cattran
D
, et al; NefIgArd Study Steering Committee. Why target the gut to treat IgA nephropathy?
Kidney Int Rep
2020;5(10):1620–1624.33102954
10.1016/j.ekir.2020.08.009PMC756968917

Wang
JJ
, 
Xu
Y
, 
Liu
FF
, et al. Association of the infectious triggers with childhood Henoch‐Schonlein purpura in Anhui province, China. J Infect Public Health
2020;13(1):110–117.31337540
10.1016/j.jiph.2019.07.00418

Hwang
HH
, 
Lim
IS
, 
Choi
BS
, et al. Analysis of seasonal tendencies in pediatric Henoch‐Schönlein purpura and comparison with outbreak of infectious diseases. Medicine (Baltimore)
2018;97(36):e12217.30200139
10.1097/MD.0000000000012217PMC613364419

Mossberg
M
, 
Segelmark
M
, 
Kahn
R
, et al. Epidemiology of primary systemic vasculitis in children: a population‐based study from southern Sweden. Scand J Rheumatol
2018;47(4):295–302.29409373
10.1080/03009742.2017.141249720

Maisons
V
, 
Ramdani
Y
, 
Hankard
A
, et al. New insights into epidemiological data and impact of the COVID‐19 pandemic on IgA vasculitis in children and adults: a French nationwide cohort. Rheumatol Int
2023;43(10):1791–1798.37438546
10.1007/s00296-023-05387-221

Nossent
JC
, 
Raymond
W
, 
Keen
H
, et al. Infection rates before and after diagnosis of IgA vasculitis in childhood: a population‐wide study using non‐exposed matched controls. J Rheumatol
2020;47(3):424–430.31203216
10.3899/jrheum.19011022

Rasmussen
C
, 
Tisseyre
M
, 
Garon‐Czmil
J
, et al. Drug‐induced IgA vasculitis in children and adults: revisiting drug causality using a dual pharmacovigilance‐based approach. Autoimmun Rev
2021;20(1):102707.33197572
10.1016/j.autrev.2020.10270723

Suszek
D
, 
Grzywa‐Celińska
A
, 
Emeryk‐Maksymiuk
J
, et al. IgA vasculitis after COVID‐19: a case‐based review. Rheumatol Int
2024;44(7):1353–1357.38739223
10.1007/s00296-024-05606-4PMC1117859624

Sugino
H
, 
Sawada
Y
, 
Nakamura
M
. IgA vasculitis: etiology, treatment, biomarkers and epigenetic changes. Int J Mol Sci
2021;22(14):7538.34299162
10.3390/ijms22147538PMC830794925

Di Vincenzo
F
, 
Ennas
S
, 
Pizzoferrato
M
, et al. Henoch‐Schonlein purpura following exposure to SARS‐CoV2 vaccine or infection: a systematic review and a case report. Intern Emerg Med
2024;19(1):13–37.37500944
10.1007/s11739-023-03366-wPMC1082783526

Ramdani
Y
, 
Largeau
B
, 
Jonville‐Bera
AP
, et al. COVID‐19 vaccination as a trigger of IgA vasculitis: a global pharmacovigilance study. J Rheumatol
2023;50(4):564–567.36379583
10.3899/jrheum.22062927

Umeda
C
, 
Fujinaga
S
, 
Endo
A
, et al. Preventive effect of tonsillectomy on recurrence of Henoch‐Schönlein Purpura nephritis after intravenous methylprednisolone pulse therapy. Tohoku J Exp Med
2020;250(1):61–69.31996498
10.1620/tjem.250.6128

Kanai
H
, 
Sawanobori
E
, 
Kobayashi
A
, et al. Early treatment with methylprednisolone pulse therapy combined with tonsillectomy for heavy proteinuric Henoch‐Schönlein purpura nephritis in children. Nephron Extra
2011;1(1):101–111.22470384
10.1159/000333010PMC329084029

Gardner‐Medwin
JMM
, 
Dolezalova
P
, 
Cummins
C
, et al. Incidence of Henoch‐Schönlein purpura, Kawasaki disease, and rare vasculitides in children of different ethnic origins. Lancet
2002;360(9341):1197–1202.12401245
10.1016/S0140-6736(02)11279-730

Gupta
V
, 
Aggarwal
A
, 
Gupta
R
, et al. Differences between adult and pediatric onset Henoch‐Schonlein purpura from North India. Int J Rheum Dis
2018;21(1):292–298.29115055
10.1111/1756-185X.1322131

Harris
BW
, 
Maxfield
L
, 
Hunter
A
, et al. Worldwide distribution and extracutaneous manifestations of Henoch‐Schönlein purpura in adults: narrative review. JMIR Dermatol
2024;7:e49746.38271008
10.2196/49746PMC1085385832

Jelusic
M
, 
Sestan
M
. IgA vasculitis or Henoch‐Schönlein purpura: genetics and beyond. Pediatr Nephrol
2021;36(8):2149–2153.33591408
10.1007/s00467-021-04987-z33

Smith
CIE
, 
Bergman
P
, 
Hagey
DW
. Estimating the number of diseases ‐ the concept of rare, ultra‐rare, and hyper‐rare. iScience
2022;25(8):104698.35856030
10.1016/j.isci.2022.104698PMC928759834

Ozen
S
, 
Pistorio
A
, 
Iusan
SM
, et al; Paediatric Rheumatology International Trials Organisation (PRINTO). EULAR/PRINTO/PRES criteria for Henoch‐Schönlein purpura, childhood polyarteritis nodosa, childhood Wegener granulomatosis and childhood Takayasu arteritis: Ankara 2008. Part II: Final classification criteria. Ann Rheum Dis
2010;69(5):798–806.20413568
10.1136/ard.2009.11665735

Audemard‐Verger
A
, 
Pillebout
E
, 
Baldolli
A
, et al. Impact of aging on phenotype and prognosis in IgA vasculitis. Rheumatology (Oxford)
2021;60(9):4245–4251.33410479
10.1093/rheumatology/keaa92136

Reamy
BV
, 
Williams
PM
, 
Lindsay
TJ
. Henoch‐Schönlein purpura. Am Fam Physician
2009;80(7):697–704.1981734037

Du
L
, 
Wang
P
, 
Liu
C
, et al. Multisystemic manifestations of IgA vasculitis. Clin Rheumatol
2021;40(1):43–52.32557258
10.1007/s10067-020-05166-538

Jauhola
O
, 
Ronkainen
J
, 
Koskimies
O
, et al. Clinical course of extrarenal symptoms in Henoch‐Schonlein purpura: a 6‐month prospective study. Arch Dis Child
2010;95(11):871–876.20371584
10.1136/adc.2009.16787439

Buscatti
IM
, 
Casella
BB
, 
Aikawa
NE
, et al. Henoch‐Schönlein purpura nephritis: initial risk factors and outcomes in a Latin American tertiary center. Clin Rheumatol
2018;37(5):1319–1324.29330742
10.1007/s10067-017-3972-340

Jauhola
O
, 
Ronkainen
J
, 
Koskimies
O
, et al. Renal manifestations of Henoch‐Schonlein purpura in a 6‐month prospective study of 223 children. Arch Dis Child
2010;95(11):877–882.20852275
10.1136/adc.2009.18239441

Ozturk
K
, 
Cakan
M
. Initial manifestations and short term follow‐up results of Henoch‐Schönlein purpura in children: a report from two centers. North Clin Istanb
2020;7(4):341–347.33043258
10.14744/nci.2019.40370PMC752109942

Avcı
B
, 
Kurt
T
, 
Aydın
F
, et al. Association of Pediatric Vasculitis Activity Score with immunoglobulin A vasculitis with nephritis. Pediatr Nephrol
2023;38(3):763–770.35895124
10.1007/s00467-022-05675-243

Karadağ
ŞG
, 
Tanatar
A
, 
Sönmez
HE
, et al. The clinical spectrum of Henoch‐Schönlein purpura in children: a single‐center study. Clin Rheumatol
2019;38(6):1707–1714.30734116
10.1007/s10067-019-04460-144

Wang
H
, 
Das
L
, 
Hoh
SF
, et al. Urinalysis monitoring in children with Henoch‐Schönlein purpura: is it time to revise?
Int J Rheum Dis
2019;22(7):1271–1277.30896086
10.1111/1756-185X.1355245

Schinzel
V
, 
Fernandez
JD
, 
Clemente
G
, et al. The profile and clinical outcomes of patients with renal involvement due to IgA vasculitis: is azathioprine a good option for treatment?
Adv Rheumatol
2019;59(1):21.31113470
10.1186/s42358-019-0064-x46

Yazılıtaş
F
, 
Çakıcı
EK
, 
Kurt Şükür
ED
, et al. Clinical spectrum of immunoglobulin A vasculitis in children and determining the best timing of urine examination to predict renal involvement. Postgrad Med
2022;134(4):441–447.35354357
10.1080/00325481.2022.206116547

Zhao
YL
, 
Liu
ZJ
, 
Bai
XM
, et al. Obesity increases the risk of renal involvement in children with Henoch‐Schönlein purpura. Eur J Pediatr
2015;174(10):1357–1363.25899072
10.1007/s00431-015-2547-z48

Carucci
NS
, 
La Barbera
G
, 
Peruzzi
L
, et al. Time of onset and risk factors of renal involvement in children with Henoch‐Schönlein purpura: retrospective study. Children (Basel)
2022;9(9):1394.36138703
10.3390/children9091394PMC949790049

AlKhater
SA
, 
Al Moaigel
HM
. Clinical spectrum and outcome of immunoglobulin A vasculitis in children: a 10‐year clinical study. Int J Clin Pract
2021;75(4):e13930.33319433
10.1111/ijcp.1393050

Kang
Y
, 
Park
JS
, 
Ha
YJ
, et al. Differences in clinical manifestations and outcomes between adult and child patients with Henoch‐Schönlein purpura. J Korean Med Sci
2014;29(2):198–203.24550645
10.3346/jkms.2014.29.2.198PMC392399751

Shin
JI
, 
Lee
SJ
, 
Lee
JS
, et al. Intravenous dexamethasone followed by oral prednisolone versus oral prednisolone in the treatment of childhood Henoch‐Schönlein purpura. Rheumatol Int
2011;31(11):1429–1432.20464400
10.1007/s00296-010-1507-152

Tabel
Y
, 
Inanc
FC
, 
Dogan
DG
, et al. Clinical features of children with Henoch‐Schonlein purpura: risk factors associated with renal involvement. Iran J Kidney Dis
2012;6(4):269–274.22797096
53

Watson
L
, 
Richardson
ARW
, 
Holt
RCL
, et al. Henoch Schonlein purpura‐‐a 5‐year review and proposed pathway. PLoS One
2012;7(1):e29512.22235302
10.1371/journal.pone.0029512PMC325043454

Çakıcı
EK
, 
Gür
G
, 
Yazılıtaş
F
, et al. A retrospective analysis of children with Henoch‐Schonlein purpura and re‐evaluation of renal pathologies using Oxford classification. Clin Exp Nephrol
2019;23(7):939–947.30895528
10.1007/s10157-019-01726-555

Sestan
M
, 
Srsen
S
, 
Kifer
N
, et al. Persistence and severity of cutaneous manifestations in IgA vasculitis is associated with development of IgA vasculitis nephritis in children. Dermatology
2022;238(2):340–346.34098552
10.1159/00051676556

Sestan
M
, 
Kifer
N
, 
Sozeri
B
, et al; Vasculitis Working Party of the Pediatric Rheumatology European Society (PReS). Clinical features, treatment and outcome of pediatric patients with severe cutaneous manifestations in IgA vasculitis: multicenter international study. Semin Arthritis Rheum
2023;61:152209.37126983
10.1016/j.semarthrit.2023.15220957

Sunar Yayla
EN
, 
Bakkaloğlu
SA
. Does age at disease onset affect the clinical presentation and outcome in children with immunoglobulin A vasculitis?
Arch Rheumatol
2023;38(4):633–641.38125056
10.46497/ArchRheumatol.2023.9914PMC1072874858

Zheng
X
, 
Chen
Q
, 
Chen
L
. Obesity is associated with Henoch‐Schönlein purpura nephritis and development of end‐stage renal disease in children. Ren Fail
2019;41(1):1016–1020.31735105
10.1080/0886022X.2019.1685545PMC688247559

Karadağ
ŞG
, 
Çakmak
F
, 
Çil
B
, et al. The relevance of practical laboratory markers in predicting gastrointestinal and renal involvement in children with Henoch‐Schönlein purpura. Postgrad Med
2021;133(3):272–277.32772751
10.1080/00325481.2020.180716160

Cao
T
, 
Yang
HM
, 
Huang
J
, et al. Risk factors associated with recurrence of Henoch‐Schonlein purpura: a retrospective study. Front Pediatr
2023;11:1164099.37377759
10.3389/fped.2023.1164099PMC1029160961

Gómez
S
, 
Pérez
M
, 
Pellegrini
M
, et al. Henoch‐Schonlein purpura in pediatrics: ten years of experience at a moderate risk office of a general hospital. Arch Argent Pediatr
2020;118(1):31–37.31984693
10.5546/aap.2020.eng.3162

Kelly
BG
, 
Stratton
DB
, 
Mansour
I
, et al. Navigating the initial diagnosis and management of adult IgA vasculitis: a review. JAAD Int
2022;8:71–78.35721303
10.1016/j.jdin.2022.05.004PMC920472963

Kurnia
B.

Henoch‐Schonlein purpura in children: the role of corticosteroids. Open Access Maced J Med Sci
2019;7(11):1812–1814.31316664
10.3889/oamjms.2019.538PMC661427264

Williams
CEC
, 
Lamond
M
, 
Marro
J
, et al. A narrative review of potential drug treatments for nephritis in children with IgA vasculitis (HSP). Clin Rheumatol
2023;42(12):3189–3200.37755547
10.1007/s10067-023-06781-8PMC1064047865

Hahn
D
, 
Hodson
EM
, 
Craig
JC
. Interventions for preventing and treating kidney disease in IgA vasculitis. Cochrane Database Syst Rev
2023(2):CD005128.36853224
10.1002/14651858.CD005128.pub4PMC997277766

Oni
L
, 
Platt
C
, 
Marlais
M
, et al. National recommendations for the management of children and young people with IgA vasculitis: a best available evidence, group agreement‐based approach. Arch Dis Child
2025;110:67–76.10.1136/archdischild-2024-327364PMC116719973937913967

Rovin
BH
, 
Adler
SG
, 
Barratt
J
, et al. Executive summary of the KDIGO 2021 guideline for the management of glomerular diseases. Kidney Int
2021;100(4):753–779.34556300
10.1016/j.kint.2021.05.01568

Rauen
T
, 
Eitner
F
, 
Fitzner
C
, et al; STOP‐IgAN Investigators. Intensive supportive care plus immunosuppression in IgA nephropathy. N Engl J Med
2015;373(23):2225–2236.26630142
10.1056/NEJMoa141546369

Ronkainen
J
, 
Koskimies
O
, 
Ala‐Houhala
M
, et al. Early prednisone therapy in Henoch‐Schönlein purpura: a randomized, double‐blind, placebo‐controlled trial. J Pediatr
2006;149(2):241–247.16887443
10.1016/j.jpeds.2006.03.02470

Trivioli
G
, 
Fenoglio
R
, 
Pillebout
E
, et al. Rituximab in adult‐onset IgA vasculitis [abstract]. Nephrol Dial Transplant
2024;39(suppl 1):i238–i239.71

Jelusic
M
, 
Sestan
M
, 
Giani
T
, et al. New insights and challenges associated with IgA vasculitis and IgA vasculitis with nephritis‐is it time to change the paradigm of the most common systemic vasculitis in childhood?
Front Pediatr
2022;10:853724.35372148
10.3389/fped.2022.853724PMC896528372

Lafayette
R
, 
Kristensen
J
, 
Stone
A
, et al; NefIgArd trial investigators. Efficacy and safety of a targeted‐release formulation of budesonide in patients with primary IgA nephropathy (NefIgArd): 2‐year results from a randomised phase 3 trial. Lancet
2023;402(10405):859–870.37591292
10.1016/S0140-6736(23)01554-473

Davies
E
, 
Chetwynd
A
, 
McDowell
G
, et al. The current use of proteomics and metabolomics in glomerulonephritis: a systematic literature review. J Nephrol
2024;37(5):1209–1225.38689160
10.1007/s40620-024-01923-wPMC1140544074

Hočevar
A
, 
Rotar
Z
, 
Jurčić
V
, et al. IgA vasculitis in adults: the performance of the EULAR/PRINTO/PRES classification criteria in adults. Arthritis Res Ther
2016;18(1):58.26935833
10.1186/s13075-016-0959-4PMC477414375

Kiryluk
K
, 
Sanchez‐Rodriguez
E
, 
Zhou
XJ
, et al. Genome‐wide association analyses define pathogenic signaling pathways and prioritize drug targets for IgA nephropathy. Nat Genet
2023;55(7):1091–1105.37337107
10.1038/s41588-023-01422-xPMC1182468776

López‐Mejías
R
, 
Carmona
FD
, 
Castañeda
S
, et al. A genome‐wide association study suggests the HLA class II region as the major susceptibility locus for IgA vasculitis. Sci Rep
2017;7(1):5088.28698626
10.1038/s41598-017-03915-2PMC550600277

Koskela
M
, 
Nihtilä
J
, 
Ylinen
E
, et al. HLA‐DQ and HLA‐DRB1 alleles associated with Henoch‐Schönlein purpura nephritis in Finnish pediatric population: a genome‐wide association study. Pediatr Nephrol
2021;36(8):2311–2318.33591409
10.1007/s00467-021-04955-7PMC826052878

Xia
L
, 
Chen
M
, 
Zhang
H
, et al. Genome‐wide association study of 7661 Chinese Han individuals and fine‐mapping major histocompatibility complex identifies HLA‐DRB1 as associated with IgA vasculitis. J Clin Lab Anal
2022;36(6):e24457.35470498
10.1002/jcla.24457PMC916916279

López‐Mejías
R
, 
Genre
F
, 
Pérez
BS
, et al. Association of HLA‐B*41:02 with Henoch‐Schönlein Purpura (IgA Vasculitis) in Spanish individuals irrespective of the HLA‐DRB1 status. Arthritis Res Ther
2015;17(1):102.25889603
10.1186/s13075-015-0622-5PMC441639180

Amoli
MM
, 
Thomson
W
, 
Hajeer
AH
, et al. HLA‐B35 association with nephritis in Henoch‐Schönlein purpura. J Rheumatol
2002;29(5):948–949.12022355
81

Aggarwal
R
, 
Gupta
A
, 
Naru
J
, et al. HLA‐DRB1 in Henoch‐Schönlein purpura: a susceptibility study from North India. Hum Immunol
2016;77(7):555–558.27184863
10.1016/j.humimm.2016.05.00982

Soylemezoglu
O
, 
Peru
H
, 
Gonen
S
, et al. CTLA‐4 +49 A/G genotype and HLA‐DRB1 polymorphisms in Turkish patients with Henoch‐Schönlein purpura. Pediatr Nephrol
2008;23(8):1239–1244.18449568
10.1007/s00467-008-0837-783

Liu
L
, 
Zhu
L
, Monteiro‐Martins S, et al. Genome‐wide studies define new genetic mechanisms of IgA vasculitis. medRxiv Preprint posted online October 11, 2024. doi:10.1101/2024.10.10.24315041
84

López‐Mejías
R
, 
Castañeda
S
, 
Genre
F
, et al. Genetics of immunoglobulin‐A vasculitis (Henoch‐Schönlein purpura): an updated review. Autoimmun Rev
2018;17(3):301–315.29353097
10.1016/j.autrev.2017.11.02485

Friedrich
J
, 
Bellmann
M
, 
Klank
D
, et al. Clinical and histological comparison of IgA nephritis and renal IgA vasculitis. Nephrol Dial Transplant
2024;40(1):182–192.38908911
10.1093/ndt/gfae14386

Kiryluk
K
, 
Moldoveanu
Z
, 
Sanders
JT
, et al. Aberrant glycosylation of IgA1 is inherited in both pediatric IgA nephropathy and Henoch‐Schönlein purpura nephritis. Kidney Int
2011;80(1):79–87.21326171
10.1038/ki.2011.16PMC364156187

Yu
S
, 
Zhang
R
, 
Lin
Y
, et al. Blood soluble CD89‐IgA complex may be a potential biomarker for predicting multi‐organ involvement, especially renal involvement in children with immunoglobulin A vasculitis. Int Immunopharmacol
2024;142(Pt A):113063.39241523
10.1016/j.intimp.2024.11306388

Kung
VL
, 
Avasare
R
, 
Friedman
MA
, et al. Targeted transcriptional analysis of IgA vasculitis, IgA nephropathy, and IgA‐dominant infection‐related glomerulonephritis reveals both distinct and overlapping immune signatures. Kidney360
2023;4(6):e759–e768.37036681
10.34067/KID.0000000000000123PMC1037137889

Xu
L
, 
Gan
T
, 
Chen
P
, et al. Clinical application of polygenic risk score in IgA nephropathy. Phenomics
2024;4(2):146–157.38884057
10.1007/s43657-023-00138-6PMC1116931390

Pillebout
E
, 
Jamin
A
, 
Ayari
H
, et al; HSPrognosis group. Biomarkers of IgA vasculitis nephritis in children. PLoS One
2017;12(11):e0188718.29190714
10.1371/journal.pone.0188718PMC570880091

Berthelot
L
, 
Jamin
A
, 
Viglietti
D
, et al; HSPrognosis Group; members of the HSPrognosis Group. Value of biomarkers for predicting immunoglobulin A vasculitis nephritis outcome in an adult prospective cohort. Nephrol Dial Transplant.
2018;33(9):1579–1590.29126311
10.1093/ndt/gfx30092

Williams
CEC
, 
Toner
A
, 
Wright
RD
, et al. A systematic review of urine biomarkers in children with IgA vasculitis nephritis. Pediatr Nephrol
2021;36(10):3033–3044.33993342
10.1007/s00467-021-05107-7PMC844586093

Pillebout
E.

IgA vasculitis and IgA nephropathy: same disease?
J Clin Med
2021;10(11):2310.34070665
10.3390/jcm10112310PMC819779294

Selewski
DT
, 
Ambruzs
JM
, 
Appel
GB
, et al; CureGN Consortium
. Clinical characteristics and treatment patterns of children and adults with IgA nephropathy or IgA vasculitis: findings from the CureGN study. Kidney Int Rep
2018;3(6):1373–1384.30450464
10.1016/j.ekir.2018.07.021PMC622461995

Trimarchi
H
, 
Barratt
J
, 
Cattran
DC
, et al; IgAN Classification Working Group of the International IgA Nephropathy Network and the Renal Pathology Society; Conference Participants. Oxford classification of IgA nephropathy 2016: an update from the IgA Nephropathy Classification Working Group. Kidney Int
2017;91(5):1014–1021.28341274
10.1016/j.kint.2017.02.00396

Gipson
DS
, 
Selewski
DT
, 
Massengill
SF
, et al. NephCure Accelerating Cures Institute: a multidisciplinary consortium to improve care for nephrotic syndrome. Kidney Int Rep
2017;3(2):439–446.29725648
10.1016/j.ekir.2017.11.016PMC593213397

Mayer‐Hain
S
, 
Gebhardt
K
, 
Neufeld
M
, et al. Systemic activation of neutrophils by immune complexes is critical to IgA vasculitis. J Immunol
2022;209(6):1048–1058.35985788
10.4049/jimmunol.210092498

Qin
J
, 
Zhang
L
, 
Ke
B
, et al. Causal relationships between circulating inflammatory factors and IgA vasculitis: a bidirectional Mendelian randomization study. Front Immunol
2023;14:1248325.37753071
10.3389/fimmu.2023.1248325PMC1051851799

Chen
XQ
, 
Zou
JS
, 
Tu
L
, et al. Neutrophil extracellular traps involved in the pathogenesis of IgA vasculitis: confirmed in two IgAV rat models. PLoS One
2023;18(7):e0288538.37478141
10.1371/journal.pone.0288538PMC10361466100

Hernández‐Rodríguez
J
, 
Carbonell
C
, 
Mirón‐Canelo
JA
, et al. Rituximab treatment for IgA vasculitis: a systematic review. Autoimmun Rev
2020;19(4):102490.32062030
10.1016/j.autrev.2020.102490

## Supporting information


**Disclosure form**.
